# Wind Environment Simulation Accuracy in Traditional Villages with Complex Layouts Based on CFD

**DOI:** 10.3390/ijerph18168644

**Published:** 2021-08-16

**Authors:** Xingbo Yao, Shuo Han, Bart Dewancker

**Affiliations:** 1 Faculty of Environmental Engineering, The University of Kitakyushu, Kitakyushu 808-0135, Japan; bart@kitakyu-u.ac.jp; 2 School of Communication Engineering, Xidian University, Xi’an 710071, China; hanshuo1991sure@163.com

**Keywords:** CFD, building environment, steady-state simulation, rural ventilation

## Abstract

Using wind speed, wind direction, and turbulence intensity values as evaluation indicators, the ventilation performance of villages with complex building layouts was studied. We used the SKE, RNG, and RKE solvers in CFD-3D steady-state Reynolds-averaged Navier–Stokes (RANS) to simulate the wind environment of a village. The findings show that for the simulation of rural wind environments with complex building layouts, steady-state simulation solvers need to be evaluated in detail to verify their accuracy. In this study, a village with a complex architectural layout in Southern Shaanxi, China, was taken as the research object, and three steady-state simulation solvers were used to evaluate the ventilation performance of the village. The simulated data were compared with the measured data to find the most suitable solver for this kind of village wind environment simulation. The results show that for the simulation of the village wind environment with a complex building layout, the RNG simulation results have the lowest reliability among the three steady-state solvers. The reliability of wind speed distribution and turbulence intensity distribution are 0.7881 and 0.2473, respectively. However, the wind speed and turbulence intensity values obtained by the SKE solver are the closest to the measured values, which are 0.8625 and 0.9088, respectively. Therefore, for villages with complex building layouts, the SKE solver should be the first choice for simulating wind environment distribution. When using the RNG solver, the overall turbulence intensity value obtained is higher than the measured value. The average deviation between the simulated data and SKE and RKE at a height of 1.7 m is 42.61%. The main reason for this is that RNG overestimates the vortices and underestimates the airflow rate in the building intervals.

## 1. Introduction

### 1.1. Motivation

Worldwide, the rural population accounts for 46% of the total population but is expected to decrease to 34% by 2050 [1]. In China, with the rapid increase in rural urbanization, problems such as urban heat islands and reduced air quality have gradually appeared in some rural areas. Air quality has become one of the key concerns of scholars in China and throughout Asia. According to a survey, if corresponding mitigation measures are not implemented at this stage, outdoor air pollution will be the main inducing factor leading to premature and abnormal death of humans by 2050 [2]. The global urban air pollution coverage rate has exceeded 80%, and this polluted air is spreading from cities to rural areas [3]. However, the treatment of rural air pollution is a complicated and long process, requiring large amounts of human resources and funding. Therefore, reasonably improving the ventilation performance of villages using natural ventilation and the natural surrounding environment as much as possible, and improving the microclimate of villages and towns to improve their air quality have become research hotspots in recent years. To improve the air quality in a village, it is first necessary to evaluate its air quality to accurately understand the rural ventilation status, to formulate a reasonable governance strategy.

Urban ventilation is also called urban air permeability, which refers to the uninterrupted exchange of air around and above a city that has air pollution, through which the concentration of air pollutants is diluted, air temperature is reduced, and air humidity is improved [4]. The reduction in air pollution by changing the city’s ventilation performance was verified in previous studies, and it plays a vital role in the improvement in urban air quality [5]. The concept of urban ventilation is also applicable to villages and towns, where its effect is even more obvious than in cities. The natural environment around a village is superior to that of a city, so it is easier to use the inherent natural conditions to improve the microclimate of a village. The ventilation performance of a village is not only determined by various aspects of the external environment, such as wind speed, wind direction, external environmental temperature, air humidity, etc., but also by the inherent geometric parameters of the village, such as the density of the buildings [6], aspect ratio [7], street scale, etc. [8]. In addition, the degree of air pollution is related to the distance between the pollution source and the village, the size of the pollution source, the emission characteristics, and the compactness of the village or town [9].

In previous studies, the research methods used for the analysis of urban ventilation mainly included field measurements and indoor wind tunnel experiments [10]. The importance of these methods in fluid mechanics has been confirmed in many studies. These two methods are impeccable for the presentation of data accuracy, but they are time-consuming, the number of instruments that can be used is limited, and they excessively consume human resources. However, with the gradual development and maturity of computer science in recent years, computer simulation has been used as a new research method for fluid dynamics. This research method is maturing and can be applied in many research fields, such as wind and building interaction, fire prevention, pollutant diffusion prediction [11], outdoor village comfort [12], indoor and outdoor wind environment, and thermal environment [13], convective heat transfer [14], etc. Computer simulation can not only be used to effectively solve the problems of the insufficient number of instruments and the need for human resources when extracting wind environment data for large cities and towns but also allows all operations to be completed by computers, increasing the convenience and speed of the whole process. However, despite pursuing convenience and speed, the accuracy of the simulation must be ensured. Many factors affect the accuracy of simulation, including the complexity of the layout of villages and towns, the accuracy of the research model, the setting of various parameters, and the choice of simulation methods. Most previous studies were based on the analysis of the wind environment by simplifying the town model to simple geometric shapes. A few studies on the arrangement of complex buildings only focused on cities [15]. No fixed simulation method for outdoor ventilation in the countryside has been developed. Some scholars studied the choice of transient and steady-state [16], but the overall comparison between LES and RANS involves a variety of different steady-state simulation equations, such as SKE, RNG, RKE, etc. Compared to transient simulation, steady-state simulation is faster, greatly shortening the time required for calculation and improving work efficiency. It has been widely used in various studies. However, when using steady-state simulation to simulate the wind environment of complex villages, few have analyzed the differences between these equations or which equation is more suitable for the study of the wind environment of complex villages. Therefore, in this study, firstly, a village with a complex arrangement of buildings was selected as a research case, and field wind environment data were measured in the village. Secondly, SKE, RNG, and RKE were separately used to simulate the wind environment distribution of the study area using steady-state simulation. The three simulation results were compared in terms of the horizontal direction, vertical direction, and local detail, and the deviation range of the three simulations and the causes of the deviation was discussed. Finally, the transient equation most suitable for the wind environment simulation of complex towns and villages was selected. Our findings provide certain guiding significance for the study of ventilation, the prediction of air pollution diffusion, and the analysis of outdoor thermal environments in villages.

### 1.2. Previous Studies

With the development of CFD technology, more scholars are using fluid simulation to analyze the ventilation performance of a settlement to change and re-establish the microclimate of the area. The wind inside a settlement and the surrounding wind environment can be altered to better cater to the geometric characteristics of the settlement and change the status of the area’s thermal environment. Yi et al. [17] proposed an idealized high Reynolds number porous media urban model combined with multi-scale computational fluid dynamics (CFD) to quantify urban ventilation and air permeability under geostrophic wind conditions. The air permeability of the city was evaluated based on the hourly rate of air change (ACH) and the age of the air. The contribution of five factors to urban heat islands and air quality was qualitatively and quantitatively analyzed. Chao et al. [18] developed a city-scale indoor and outdoor computational fluid dynamics (CFD) coupling model and defined a novel ventilation index to evaluate the potential of natural ventilation. First, they developed a coupled CFD model to study wind cross-ventilation in high-density cities. Secondly, six key design variables were used to generate 3840 parameter design changes for the evaluation of natural ventilation. Finally, a novel comprehensive index (indoor and outdoor interaction coupling) was constructed to evaluate the wind speed ratio between the indoor area and the outdoor reference area. Luo et al. [19] studied the parameters of the ideal city model based on CFD. Taking Tokyo, Japan; Los Angeles and Phoenix, the United States; and Hong Kong as the research objects, the potential of slope flow for urban ventilation in mountainous areas without background weather was studied.

CFD has also significantly contributed to the tracking of the diffusion path of air pollutants in the settlement and reduction of pollutant concentration. Lingjie et al. [20] proposed a new type of circulation system, using the rubber refining process as an example, to concentrate pollutants and reduce exhaust gas. A circulating ventilation model based on mass balance was established to predict the changes in pollutant concentration in the system, and two control strategies (i.e., continuous or intermittent exhaust schemes) were developed to improve the pollutant capture efficiency of the system. The emission intensity of pollutants in the rubber refining process was measured and used as a boundary condition in the subsequent simulation. CFD simulation was used to optimize the circulating air volume, return air angle, and exhaust volume of the circulating system. Lauriks et al. [21] used CFD to analyze the level and distribution of pollutants in a part of Antwerp’s main road artery (Belgium, Europe). Erfan et al. [22] investigated the influence of the cross-sectional shape of a building on the diffusion of air pollutants around the isolated building. Based on detailed wind tunnel experimental data, a CFD model was developed and verified. Compared to the RANS model, the LES model was found to have higher consistency with the experimental results, especially in terms of replicating the pollutant diffusion characteristics related to the interaction of the wind structure. Jiang et al. [23] used CFD to simulate the changes in neighborhood microclimate and pollutant diffusion distribution under different weather conditions and proposed three strategies for the optimization of urban planning to alleviate air pollution. Fabiana et al. [24] investigated the impact of different urban block types on the diffusion of urban pollutants. Using computational fluid dynamics technology, five types of real cities were studied: single, independent buildings, central courtyards, internal courtyards, and determinant layouts. Numerical simulations were conducted using the unsteady-state Reynolds-averaged Navier-Stokes (URANS) equation and the SST model to express the turbulence effect. Peng et al.

The verification of the simulation accuracy of various solvers in CFD software also plays a decisive role in the study and analysis of wind environments. Tatsuhiro et al. [25] used Hygrabe2D coupled with heat transfer simulation (HTS) to calculate indoor surface temperature, and the convection and convective heat transfer coefficients between regions are calculated using CFD and transferred to heat transfer simulation (HTS). The accuracy of the proposed method was verified. Mohammad et al. [26] focused on carefully separating patches with different thermal characteristics or directions to capture their impact on a cooling device. Their numerical simulations were verified by performing field measurements. The results clarified the key role of wind patterns in reducing or intensifying local urban heat islands (UHIs). The methods provided by Chao et al. [27] can answer questions encountered by wind energy consultants and architects, especially in terms of input boundary conditions, simulation modeling, model verification, and data collection and analysis. Hypothesis testing methods were introduced into the framework to verify and evaluate simulation results. Esther et al. [28] evaluated CFD simulation results using the measurement results provided by a city’s air quality monitoring station network and the mobile microsensor network carried by cyclists during their daily commuting. After calculating the annual average concentration of NO_2_, the maximum relative deviation between the CFD and the measured data was found to be less than 30%. Tatsuhiro et al. [29] developed a new coupling method of energy simulation (ES) and CFD, verified the effectiveness of the fixed part of the coupling method, and predicted the spatial temperature distribution. Taeyeon et al. [30] used wind tunnel experiments and CFD to analyze the ventilation performance of closed shopping malls, providing useful design information for shopping malls. The scaled-down model of the building was used in wind tunnel experiments to verify the reliability of the CFD method. Based on the structure and arcade design of the mall, 11 design alternatives were proposed, and their performance was evaluated as the air exchange rate using a proven CFD simulation. Gaber et al.

### 1.3. Scientific Originality

CFD simulation has become the most widely used research method for the study of settlement wind environment, thermal environment, and air quality. However, most of the main cases studies are cities, with analyses lacking in the rural built environment. Firstly, huge differences exist in the types, layout methods, and street and lane scales between urban and rural buildings. It is not advisable to directly apply urban building environment improvement strategies and coping methods to rural areas. Studies specifically aimed at the rural microclimate are needed to provide a theoretical basis for improving ventilation performance and alleviating air pollution. Secondly, research on the ventilation performance and air pollution of an area often require the macro-control of its overall wind environment. However, most studies on the ventilation performance of settlements are based on ideal geometric models or parts of a city. There are relatively few simulation cases of the overall wind environment of settlements with complex architectural layouts. Finally, for the accuracy of the simulation, the choice of the solver is important. Different solvers have different operation methods and characteristics. In the simulation, the solvers and calculation equations that are most suitable should be selected based on the different research purposes. Therefore, in this study, a village with a complex arrangement of buildings was selected as a study case. According to the building type, layout, and street scale of the case, a detailed model was constructed. Three different steady-state solvers (SKE, RNG, and RKE) were used to simulate the wind environment of the whole village, and we compared the different results with the measured data. Finally, the overall, partial, and vertical deviation ranges of the three steady-state solvers for rural simulations, the reasons for the deviation, and the selection methods of the solvers for different problems were obtained. Our findings provide instructive suggestions for the selection of the solver for the wind environment simulation of complex rural settlements.

### 1.4. Target of This Study

We used complex rural settlements as a study case and adopted a research method of comparing measured data with simulated data to address the following goals of this study:

- Using wind speed, direction, and turbulence intensity as reference factors, discussing the deviation in the results of the three steady-state solvers (SKE, RNG, and RKE), and finding the simulation solver with the smallest deviation;
- Analyzing the causes of deviation in the solver in terms of overall, local, and vertical directions;
- Choosing the most suitable solver for the simulation of complex rural settlement wind environments.

## 2. Methodology

The main research methods were as follows:

- Field survey: Different types of villages were collected and sorted, and representative villages relevant to the research were selected;
- Data measurement: The relevant data of these villages were measured, including village scope, building distribution, street size, wind speed, wind direction, turbulence intensity value, etc.;
- Summarize and organize the data: After the measured data were unified and integrated, the most representative data were used as the study case;
- Simulation analysis: A model was established based on the measured data, and three steady-state solvers were selected to separately simulate the wind environment of the buildings;
- Comparison of simulated and measured data: The simulated data were compared with the measured data, the deviation of the three solvers was calculated, and the causes of the deviation were analyzed;
- Conclusions: The most suitable solver for simulating the wind environment of complex rural settlements was found.

### 2.1. Investigation

**Figure 1:**
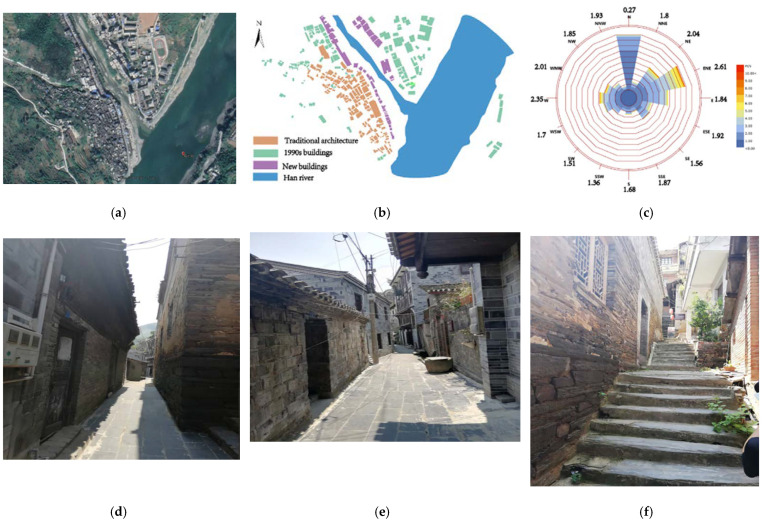
(**a**) Village satellite map. (**b**) Building age distribution. (**c**) Dominant wind direction. (**d**) Medium street. (**e**) Large streets. (**f**) Ramp street.

For this study, we selected the town of Shuhe, Ankang, Shaanxi, China. Shuhe is located in the eastern mountainous area of Xunyang County, and its administrative division is under Xunyang County, Ankang, Shaanxi. The southern border of the village is close to Baihe County, the west border is adjacent to the towns of Shuanghe and Zongxi, and the east and north borders border Yunxi County. The town area is 3.3 km^2^. Shuhe has convenient transportation, with Xiangyu Railway and National Highway No. 316 running through it. The geographic coordinates of Shuhe Town are 32°57′ N, 109°42′ E. As shown in Figure 1b, the buildings indicated by the yellow signs are traditional buildings constructed during the Qing Dynasty. Traditional buildings are mainly composed of one- and two-story buildings, of which one-story buildings account for 84.22% of the total buildings. The story height of the one-story buildings is 2.8–3 m, and that of the two-story building is 5.6–6 m. The green buildings represent buildings built in the 1990s, and the purple ones represent those built in recent years. The regional building density λp is 0.165. Figure 1c shows the wind direction and wind speed of the village. The dominant wind direction of the village is northwest, and the maximum wind speed in the middle of the year is 2.61 m/s. The terrain and landforms of Shuhe Town are complex. The village is located on a hilly landform. The overall street and lane spatial form is strongly affected by the terrain. The terrain and landform of the village largely determine the street and lane spatial forms of the village. The main streets and lanes of the village have been constructed in accordance with the contours of the mountain, and the secondary streets and lanes are connected by steps to the main streets and alleys of different heights; the overall construction method has been artificial overlap. As shown in Figure 1d,f, streets, and lanes change freely according to topography. Streets vary in width and length. Most streets and lanes are narrower, with only a few main streets and lanes reaching 5 m in width, while other laneways are mostly 2 to 3 m in width. However, for a complex and narrow road network, if we want to use CFD to simulate street ventilation, it is particularly important to choose a suitable simulation method.

### 2.2. Data Measurement and Model Setting

**Figure 2:**
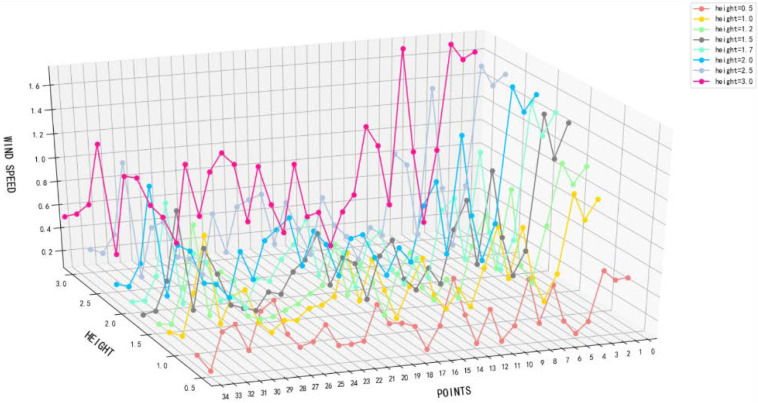
Measured wind speed values at 35 points.

**Figure 3:**
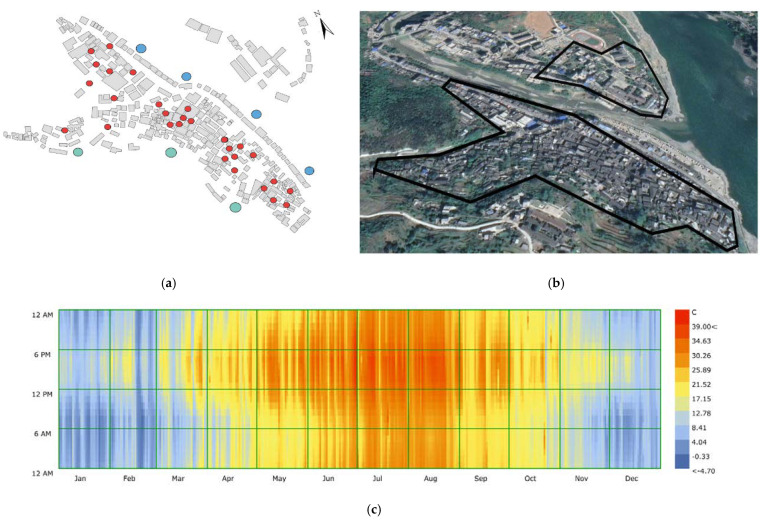

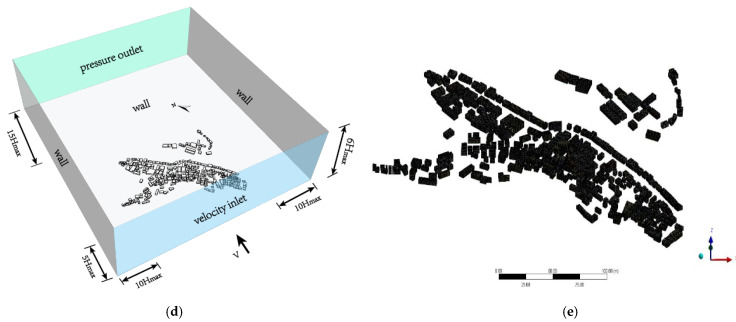
(**a**) Distribution of measuring points. (**b**) Scope of the study case. (**c**) Outdoor temperature distribution of study cases throughout the year. (**d**) Study case boundary conditions. (**e**) Meshing of study cases.

Before the field survey, the buildings in the settlement were classified according to the time of construction to understand the location of the traditional buildings in the whole settlement and to grasp the division of functional zones, so as to facilitate the data measurement in the following research. Due to its unique Qing Dynasty architectural features, Shuhe has attracted a large number of tourists from all over the country in recent years and has become a famous tourist village in Shaanxi. The core tourist area is the area with Qing Dynasty buildings. In this area, the daily flow of people is the largest (Figure 1b), so research on the ventilation performance here is particularly important. Therefore, when measuring wind speed and turbulence intensity, the distribution of Qing Dynasty buildings was regarded as the key test area. In the modern and 1990s buildings, only a few test points were selected. The distribution of test points is shown in Figure 3a. Shuhe is located on the northern edge of a subtropical zone, in a climate transition zone between China’s north and south. To the north of the village is the Qinling Mountains, blocking the cold air from the northwest to the south. The Ba Mountain in the south slows the warm and humid airflow from the southwest and southeast to the north and provides heat preservation and moisture protection. The weather in spring is changeable, with uneven hot and cold. The summer is hot, with large amounts of precipitation. It is rainy in autumn, and the river water is scarce and unstable in winter. As shown in Figure 3c, the highest temperature in the village during the year is 39.2 °C, and the lowest temperature is −4.7 °C. The annual rainfall is 800−850 mm, the average precipitation is 831.3 mm, and the relative humidity is 79%. Due to the concentrated rainfall in summer and autumn, the frequent monsoons, and the higher mountain slopes in the village, the probability of torrential rain has increased, causing the water levels of the two streams to rise, causing flood disasters. The annual average sunshine is 1790.4 h, and the maximum frozen soil depth is 0.26 m. Due to the high temperature and frequent rainfall in summer, the air humidity inside the village is directly higher. Accurate simulation of natural ventilation is particularly important for this entire village. Therefore, in this study, we chose to measure wind environment data during the peak tourist season, which has high temperatures and heavy rainfall (22–24 June). We compared the measured data with the simulated data to verify the accuracy of the three CFD model simulations. As shown in Figure 3a, a total of 35 points were selected for field measurements in the study area of the village. The test time was from 9:00 a.m. to 7:00 p.m. according to the number of tourists, and data were recorded every ten minutes. Because we mainly considered the values at the height of the tourist crowd, we focused on analyzing the height of the human body when standing and sitting. Therefore, height was controlled within the range of 0–3 m, and measured separately at 0.5, 1.0, 1.5, 1.7, 2.0, 2.5, and 3.0 m. Table 1 introduces the detailed information of the instruments used in the field measurement, and Figure 2 shows the average wind speed at the height of 0–3 m at 35 measuring points.

The calculation of turbulence intensity adopts the method provided by [31]: The turbulence intensity, also often referred to as turbulence level, is defined as:

(1) $$I=\frac{\mu^{\prime }}{U^{\prime }}$$

where $\mu^{\prime }$ is the root-mean-square of the turbulent velocity fluctuations and U is the mean velocity (Reynolds averaged).

If the turbulent energy, k, is known $\mu^{\prime }$ can be computed as:

(2) $$u^{\prime }\equiv \sqrt{\frac{1}{3}\left(u^{\prime }\frac{2}{x}+u^{\prime }\frac{2}{y}u^{\prime }\frac{2}{z}\right)\;}=\sqrt{\frac{2}{3}}k$$

U can be computed from the three mean velocity components $U_{x}$, $U_{y}$ and $U_{z}$ as:

(3) $$U=\sqrt{U_{x}^{2}+U_{y}^{2}+U_{Z}^{2}}$$

As shown in Figure 3d, the simulation geometry was established in the CFD model at a ratio of 1:1 after field measurement. The simulation mechanism of the external atmosphere is based on the methods provided in [32,33,34]. This method has undergone many experimental applications. The height was six times that of the tallest building in the village, the distance between the air outlet and the air inlet was five and fifteen times that of the tallest building, and the height and width were ten times that of the tallest building. The boundary conditions of the entrance, including the average wind speed value and turbulence intensity, were established based on the measured data, and the aerodynamic roughness length was determined by the measured average wind speed. In the inner area, an accurate model was established. The roughness of the bottom area and the wall was set to 0, which is regarded as a smooth and frictionless surface. A zero static gauge pressure was set in the exit area. The mesh was divided into a local density method (Figure 3e). The mesh used the proximity and curvature in the surface grid to divide the model into 9,357,352 units. Another advantage of using proximity and curvature is that the details inside the village can be processed more finely so that the simulated results are more accurate.

### 2.3. Setting and Verification of the Air Inlet Wind Speed

**Figure 4:**
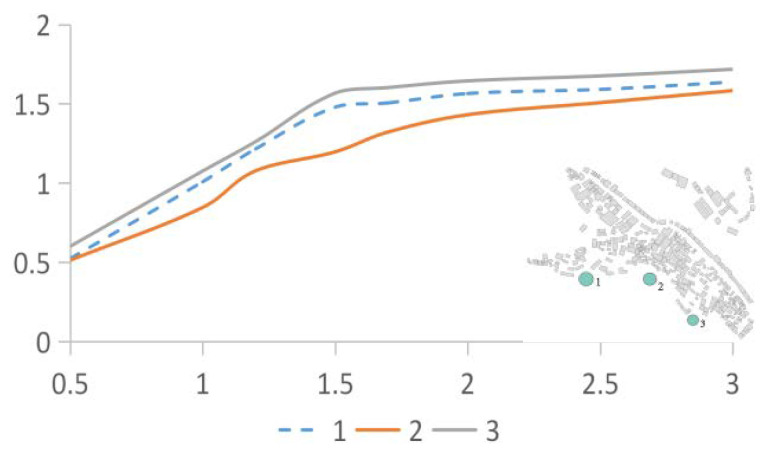
Deviation analysis of the 3 points at the air inlet.

The wind speed of the air inlet required for the simulation was determined using the measured data. As shown in Figure 3a, the average wind speed of the three green points was taken as the wind speed of the air inlet during the simulation. Before this, it was necessary to compare the wind speed at the three green points. If the wind speed difference between the three points was too large, it could not be used as the basis for the wind speed at the air inlet. Figure 4 shows the results of the comparison: the average wind speed at the height of 0.5–3.0 m at the three points at the air inlet of the study case. The difference between the No. 1 and No. 2 points is 4.635%, the difference between No. 1 and No. 3 points is 1.3%, and the difference between No. 2 and No. 3 points is 4.82%. The values are all within the deviation range, so we determined the three sets of data could be used as the basis for determining the inlet wind speed.

## 3. Results and Discussion

### 3.1. Comparison of Measured Data and Simulated Data inside the Village

**Figure 5:**
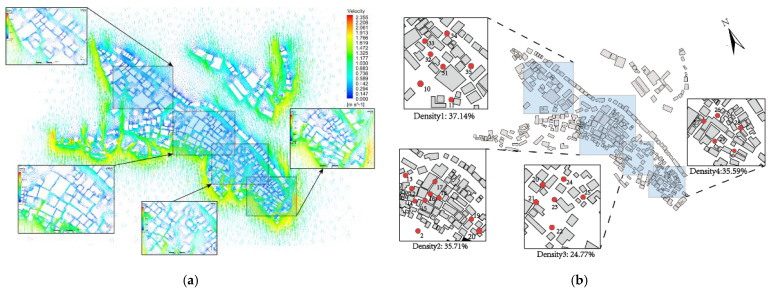
(**a**) Wind environment of study case. (**b**) Study cases are divided by different building densities.

Before conducting a detailed comparison between the measured and simulated data, we first performed a rough simulation of the overall wind environment of the village. As shown in Figure 5a, we found that the airflow rate in the village is affected by the width of the street, which depends on the building density of the area. As the data at a single measurement point may not be able to represent the characteristics of the overall wind environment in the area, the whole village was divided into four parts according to different building densities. The wind speed values of all measurement points in each area were weighted and averaged, and the calculated values were compared with the results produced by the three different solution methods to increase the representativeness of the data. As shown in Figure 5b, the building densities of the four areas are 37.14%, 35.59%, 35.71%, and 24.77%. The gradual decrease in building density, street size, and lanes in these four areas was also divided into four different levels, which decrease in sequence.

**Figure 6:**
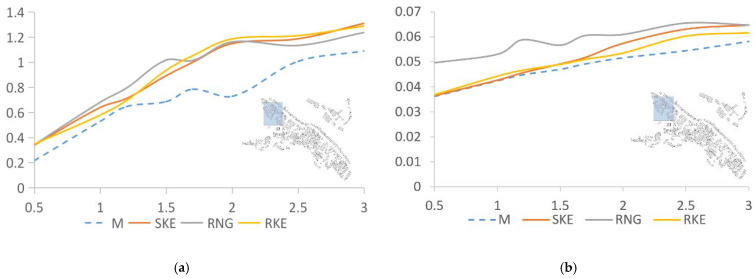
Analysis of measured and simulated data produced by SKE, RNG, and RKE in an area with a building density of 37.14%. (**a**) Wind speed comparison between SKE, RNG, RKE, and measured data; (**b**) Comparison of turbulence intensity between SKE, RNG, RKE, and measured data.

Figure 6 shows the comparative analysis of the wind speed and turbulence intensity values at seven points in the area where the building density is 37.14% and the data simulated by the three solution methods. The abscissa is the wind speed, and the ordinate is the height. The wind speed at the measuring point increases with the increase in height. After comparison, we found that different solution methods led to different deviation values between the simulated and measured data. As shown in Figure 6a, the simulation results of the three solution methods are all higher than the actual measured values. The SKE calculation result is the closest to the measured value, and the overall numerical distribution is about 9.74% higher than the measured value. The calculation results of RNG and RKE are 14.24% and 13.79% higher than the measured values, respectively. As for the turbulence intensity, Figure 6b shows that the calculation results of the three are slightly higher than the measured values, and the deviations are 11.66% and 17.2%, and 8.52%, respectively. The value simulated by RNG is significantly higher than those of SKE and RKE.

**Figure 7:**
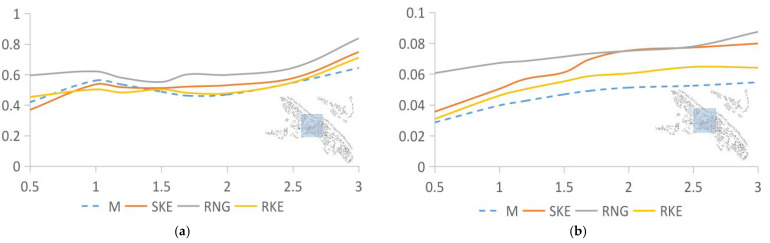
Analysis of measured values and data simulated using SKE, RNG, and RKE in an area with a building density of 35.71%. (**a**) Wind speed comparison between SKE, RNG, RKE, and the measured data. (**b**) Comparison of turbulence intensity between SKE, RNG, RKE, and the measured data.

Figure 7 compares the measured and simulated data of the 35.71% building density area, which expresses the relationship between the values simulated by SKE, RNG, and RKE and the measured values. The wind speed and turbulence intensity data solved by RNG are higher than the measured values, SKE, and RKE. As shown in Figure 7a, the wind speed data from the SKE and RKE simulations are consistent with the measured data. The deviation values are 20.53% and 26.26%, respectively. The deviation between the numerical value simulated by RNG and the measured result is 45.21%, but the movement trend of the curve is similar to the previous two. For the turbulence intensity (Figure 7b), the value produced by RNG is significantly higher than the other three curves, and the deviation is the largest, at 17.04%. The fit of the other two sets of data is above 95%, the deviation of SKE is 2.28%, and the deviation of RKE is 0.77%.

**Figure 8:**
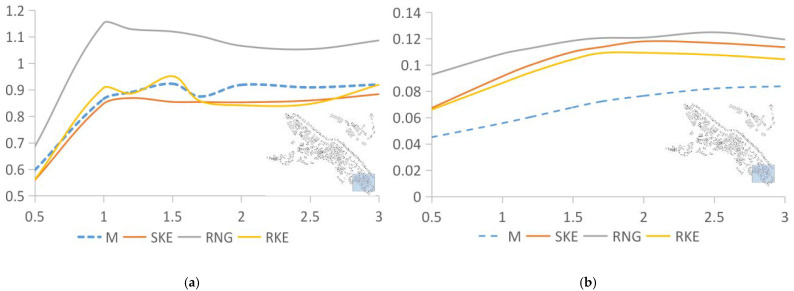
Analysis of measured and simulated data of SKE, RNG, and RKE in an area with a building density of 35.59%. (**a**) Wind speed comparison between SKE, RNG, RKE, and measured data. (**b**) Comparison of turbulence intensity between SKE, RNG, RKE, and measured data.

In the area where the building density was 35.59%, as shown in Figure 8a, the values produced by RNG are still higher than those by the other two solving methods. The data simulated by SKE and RKE are very close to the measured data. The difference between the wind speed value obtained by SKE calculation and the measured value is 3.28%, and the average distribution of wind speed is slightly lower than the measured value overall. The value obtained produced by the RNG calculation is higher overall than the measured value by 13.28%, and the value obtained by the RKE calculation is generally higher than the measured value by 21.43%. However, for turbulence intensity (Figure 8b), the results obtained by the three solution methods are consistent. Both the numerical value and the movement trend of the curve are similar to the measured values. The deviations between SKE, RNG, and RKE and the measured value are 14.1%, 17.27%, and 19.05%, respectively.

**Figure 9:**
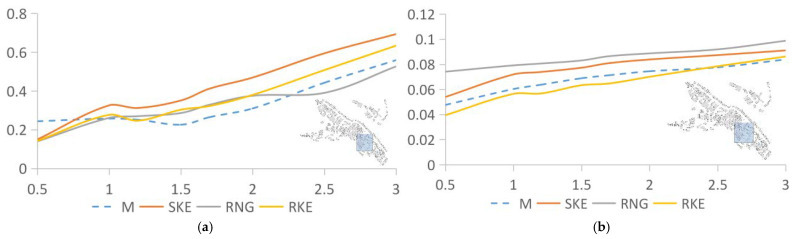
Analysis of the measured values and the values simulated by SKE, RNG, and RKE in an area with a building density of 24.77%. (**a**) Wind speed comparison between SKE, RNG, RKE, and measured data. (**b**) Comparison of turbulence intensity between SKE, RNG, RKE, and measured data.

**Figure 10:**
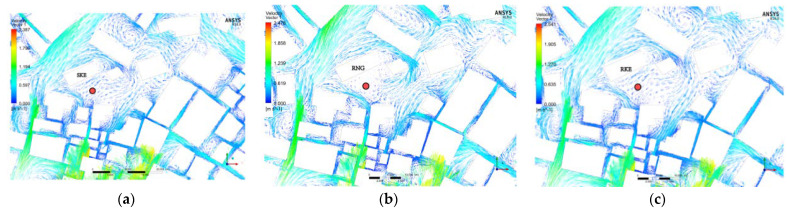
SKE-, RNG-, and RKE-simulated wind environment distribution at point 13. (**a**) SKE-simulated wind environment distribution at point 13. (**b**) RNG-simulated wind environment distribution at point 13. (**c**) RKE-simulated wind environment distribution at the point.

Figure 8 compares the measured and simulated values in the area with a 24.77% building density. When the building density is between 30% and 40%, the values produced from the RNG solution are always higher than those from SK and, RKE and the measured data. However, Figure 9a shows that when the building density drops to 24.77%, the values produced by RNG are close to the other three sets of data. Even at a height of 2–3 m, the wind speed is lower than the other three sets of data. This shows that the accuracy of the RNG solution method may be affected by building density. When the building density is higher than a certain value, using RNG will reduce the accuracy of the simulation. SKE’s deviation in the average wind speed is 20.35%, that of RNG is 26.29%, and that of RKE is 12.74%; Figure 10b depicts the turbulence intensity data and motion trends corresponding to different solutions. The three sets of simulated and measured deviations are small, 2.38%, 6.46%, and 2.68%, respectively, and the motion trends also fit well.

According to the density of the four selected areas, the deviations in the measured and simulated values of the average wind speed and turbulence intensity are listed in Table 2 and Table 3, respectively. From the data comparison, we found that the accuracy of the values simulated by RNG, whether the average wind speed or turbulence intensity, is the lowest. The deviation in the average wind speed is 24.76%. For the turbulence intensity, the deviation value is 12.33%. Conversely, in the simulation of average wind speed, the numerical deviation obtained using SKE is the smallest, at 13.47%. In the simulation value of turbulence intensity, the solution method with the smallest deviation is RKE, with a deviation value is only 6.06%. The deviation evaluation index refers to [35,36,37,38], and the error range is determined to be 15%. It can be seen that in the horizontal wind speed simulation value, only the deviation of SKE is within 15%. However, for the turbulence intensity, the three solvers are all within the deviation range.

### 3.2. Reasons for RNG Deviation

**Figure 11:**
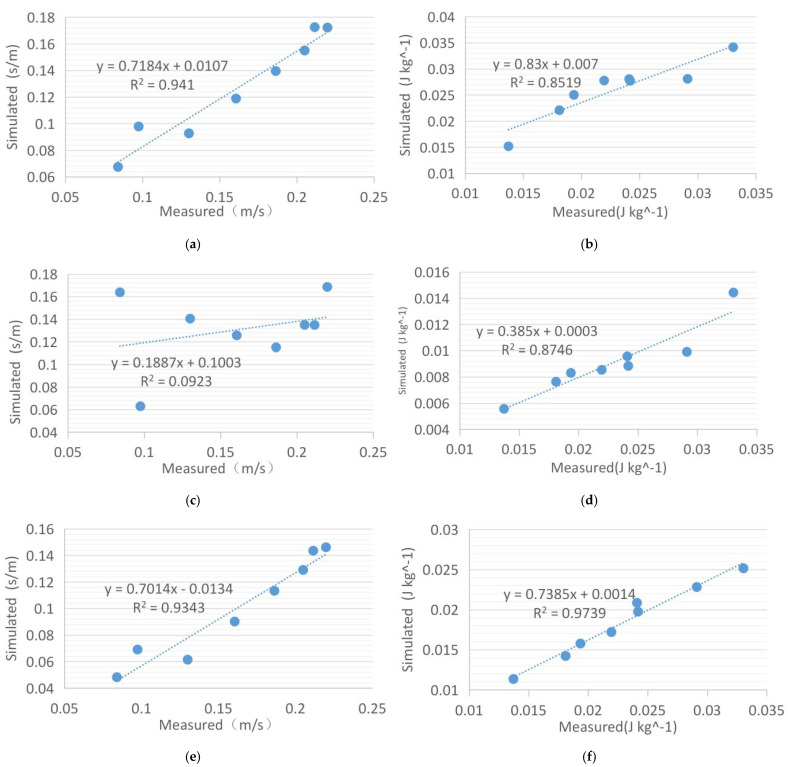
Analysis of measured and simulated data of SKE, RNG, and RKE at point 13. (**a**) Comparison of wind speed between SKE and measured data. (**b**) Comparison of turbulence intensity between SKE and measured data. (**c**) Comparison of wind speed between RNG and measured data. (**d**) Comparison of turbulence intensity between RNG and measured data. (**e**) Comparison of wind speed between RKE and measured data. (**f**) Comparison of turbulence intensity between RKE and measured data.

To determine the reason for the larger deviation in RNG, we used 3 of the 35 measuring points for further observation. These three points were points 13, 16, and 25. Taking point 13 as an example, when using SKE (Figure 10a) and RKE (Figure 10c) to solve the problem, the distribution of wind environment at point 13 is not particularly different, and the air movement trajectory is relatively smooth. However, for RNG (Figure 10b), there is a vortex at point 13 and the measurement point is located in the static pressure zone at the center of the vortex, and there is almost no airflow. Therefore, comparing the three solution methods with the measured values, the deviation of the average wind speed values of SKE and RKE are all within 15% (Figure 11a,e). However, when using RNG simulation, a large deviation occurs at these three points; as shown in Figure 11c, the simulated value and the measured value could not be fitted at all. However, for the simulated turbulence intensity (Figure 11d), the deviation produced by RNG is only 12.54%. Therefore, we assumed that the RNG solver is not accurate enough for the simulation of wind speed, but its simulation of turbulence intensity can be used as a reference.

**Figure 12:**
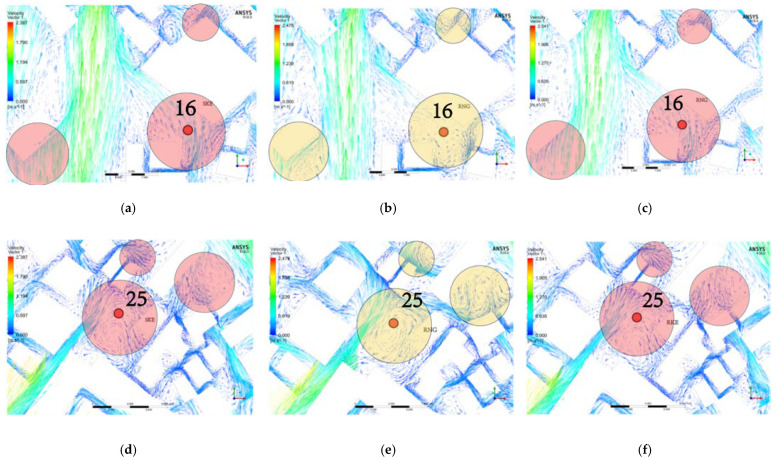
SKE-, RNG-, and RKE-simulated wind environment distribution at points 16 and 25. (**a**) SKE-simulated wind environment distribution at point 16. (**b**) RNG-simulated wind environment distribution at point 16. (**c**) RKE-simulated wind environment distribution at point 16. (**d**) SKE-simulated wind environment distribution at point 25. (**e**) RNG -simulated wind environment distribution at point 25. (**f**) RKE-simulated wind environment distribution at point 25.

Points 16 and 25 also showed the same problem. Figure 12b depicts the difference in the data from the RNG solution. The location of point 16 also has a static pressure zone, and the wind speed value approaches 0 m/s. However, with SKE and RKE, the air is flowing uniformly, and the wind speed at this point is accelerated due to the gap effect. The RNG solution results in the other two areas are significantly different from those produced by SKE and RKE. Figure 12d,f shows the wind environment distribution calculated by the three solvers around point 25. Similarly, a large number of vortices are generated in the result using RNG, and the air fluidity does not conform to the conventional logic, which is the reason for the large deviation.

### 3.3. Comparison of Wind Environment Distribution in the Overall Village

The three different solvers with different calculation methods led to differences in the simulation results. In the above, we mainly discussed the actual wind environment state at each measurement point in the village and the deviation after simulation. The values simulated by SKE and RKE are close to the measured values, but when RNG was used for simulation, a relatively large deviation occurred. Next, we discuss the comparison between the deviation generated when using the RNG solver for simulation and the use of SKE and RKE. We calculated the accuracy and deviation range of RNG’s simulation of the overall wind environment of the village.

#### 3.3.1. Horizontal Contrast

**Figure 13:**
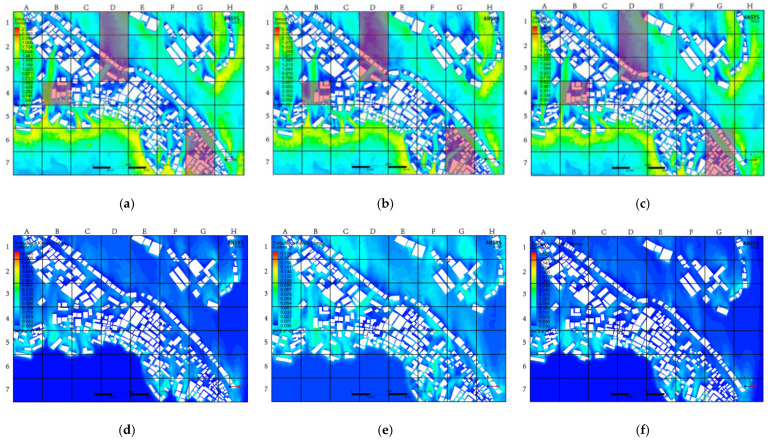
Distribution of wind environment and turbulence intensity of the whole village. (**a**) The overall village wind environment simulated by SKE. (**b**) The overall village wind environment simulated by RNG. (**c**) The overall village wind environment simulated by RKE. (**d**) Turbulence intensity of the whole village simulated by SKE. (**e**) Turbulence intensity of the whole village simulated by RNG. (**f**) Turbulence intensity of the whole village simulated by RKE.

Figure 13 shows the difference between the overall internal village wind environment layout simulated by the three solvers, and the turbulence intensity distribution in the area using the same calculation settings and parameters. In Figure 13a–c, the green area represents the active part of the airflow, and the blue area indicates that the airflow frequency is relatively low. In Figure 13d–f, the cyan area indicates that the turbulent kinetic energy is larger, and the blue area indicates that the turbulent kinetic energy is smaller. The intensity of turbulence can provide an important reference standard for expressing the age of the air; notably, in the CFD simulation of outdoor ventilation, the age of the air distribution inside the village is usually considered as the time required for the outside air to reach a specific location after entering the calculation domain [39]. Therefore, the age of the air in the study area largely depended on the definition of the inlet, and calculations were performed according to this definition. Therefore, for a complex village layout, this may cause more problems because the local average age of the air distribution in the village is more sensitive to the definition of the initial value. However, the time spent in the process of air flowing within the research range was independent of the initial value because it represents the amount of time delay caused by the airflow at each point in the village due to being blocked by buildings.

Figure 13 shows the distribution of the wind environment at a height of 1.7 m (average pedestrian height) produced by SKE, RKE, and RNG. Figure 13a–c shows the distribution of wind speed, wind direction, and wind volume in the village obtained by the three methods. To more easily compare and summarize the findings, the picture is divided into 56 squares to more accurately indicate the deviations. The distributions d (SKE) and f (RKE) shown in Figure 13 are similar, but RNG overestimates the turbulent kinetic energy, which can also be explained by RNG overestimating the air age at pedestrian height. In the red area in Figure 13a,c, the RNG simulation results obviously deviate from the other two simulations. In this case, the overall average deviation between RNG, and SKE, and RKE is about 42.61%.

#### 3.3.2. Vertical Contrast

**Figure 14:**
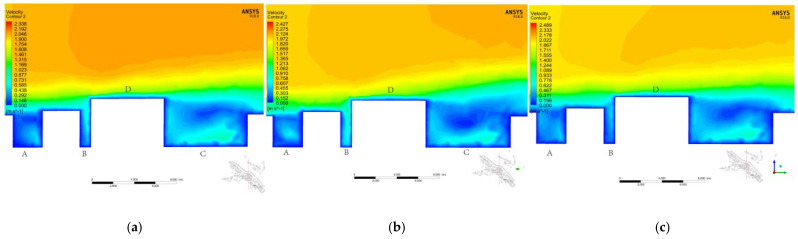

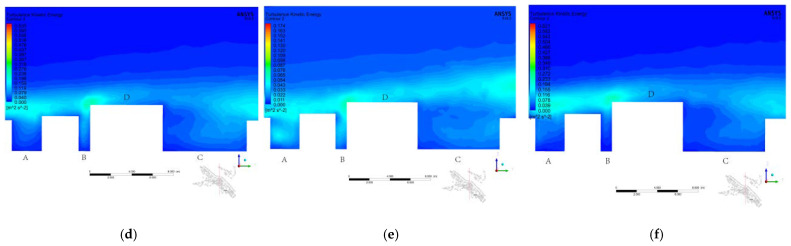
Local wind environment and turbulence intensity distribution in the vertical direction. (**a**) Local wind environment distribution simulated by SKE in the vertical direction. (**b**) Local wind environment distribution simulated by SKE in the vertical direction. (**c**) Local wind environment distribution simulated by SKE in the vertical direction. (**d**) Local wind environment distribution simulated by SKE in the vertical direction. (**e**) Local wind environment distribution simulated by SKE in the vertical direction. (**f**) Local wind environment distribution simulated by SKE in the vertical direction.

To more clearly reveal the differences between and better understand the performance of SKE, RKE, and RHG, the vertical plane at the same position in the study area was selected, and the vertical wind environment distribution and turbulence intensity distribution were determined. Figure 14a–c shows three wind speed cloud diagrams simulated by the different solvers. Figure 14d–f shows the simulation results of turbulence intensity. We found that when the wind blows on the face, the wind diverges above and around the building, causing a large amount of fresh air to enter the street along the vertical direction. This causes the average air age in the area to decrease, which could be predicted by SKE, RNG, and RKE. Notably, for SKE, RNG, and RKE, the turbulence levels in the entire roof and street canyons are well-predicted, but the turbulence can be overestimated by RNG. As shown in Figure 14d–f, at the stagnation point in front of the eaves of Building D, especially the stagnation point near the front edge, a larger separation area is produced behind Building D. For RNG, the range of the separation area is larger than those of SKE and RKE. This observation is similar to previous studies [40]. As shown in Figure 14a–c, the flow field inside Canyon B downstream of the building simulated by RNG is different from those by SKE and RKE. In addition, less turbulence sweeps the front edge of Building D and crosses the roof into the downstream area. After using RNG simulation, the turbulence level inside the canyon marked with “A” and “C” at the top of the street canyon decreased, which deviate from SKE and RKE by 43.92% and 41.54%, respectively. Figure 14a–c clearly shows that there are significant differences in the local wind environment distribution obtained by the two methods, especially in the street canyons with the “B” label. The average deviation between the stable RNG and SKE in the street canyons marked “A” and “C” is about 21.41% and 26.86%, respectively. Notably, previous studies showed that the horizontal and vertical average currents and turbulent fluctuations throughout the roof canyons significantly affect the air exchange between the street canyons and the external flows above them to help with air renewal [41].

In several examples of historical towns mainly located in the central and southern regions of China, morphological characteristics similar to those of the study area can be found. Therefore, the conclusions and any possible suggestions for improving the air permeability of the surveyed areas are considered important and may be useful to urban planners and decision-makers in these areas. Poorly ventilated areas seem to be related to lower building height changes. Therefore, increasing the variability of building heights in villages can improve air permeability in complex urban areas. This is consistent with the results of previous studies on general urban areas [42].

#### 3.3.3. Comprehensive Reliability Analysis of Actual Measurement and Simulation

The comprehensive reliability judgment method used in this study is: The index root mean square error (RMSE) [43] and coefficient of determination (R^2^) combined with python. Exponential root mean square error (RMSE) and coefficient of determination (R^2^) is used to evaluate the difference between simulated and measured values. If the RMSE error is close to zero, the most accurate model will be obtained, and a lower value indicates that the simulated value is within the measured value. Unlike RMSE, R^2^ is close to 1, and the two data are similar. The whole process of calculation is done with python.

**Figure 15:**
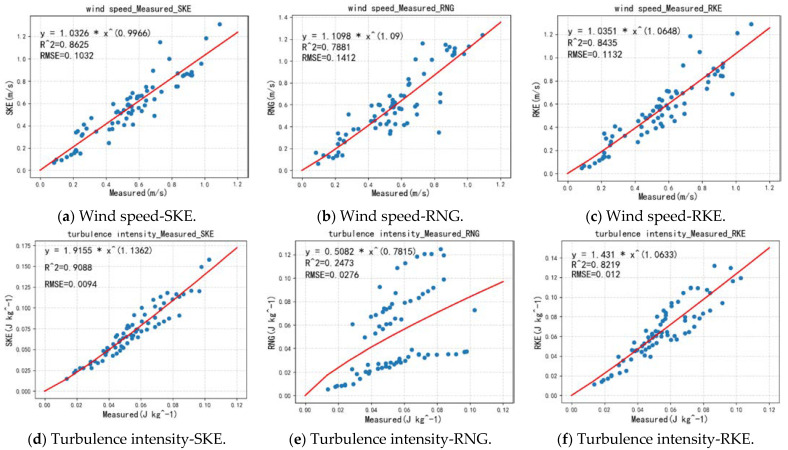
Comprehensive reliability analysis of actual measurement and simulation.

Figure 15 shows the reliability analysis of the three solvers for the simulation of the overall village wind environment. After analyzing all the horizontal and vertical data, it is found that the wind speed and turbulence intensity values simulated by SKE have the highest reliability, and the values of R^2^ are 0.8625 and 0.9088, respectively. However, whether it is wind speed or turbulence intensity, the RNG simulation results differ the most from the actual measured values, which is consistent with the previous analysis results. For wind speed, the R^2^ value of RNG is 0.7881, and for turbulence intensity, the value of R^2^ is only 0.2473. All simulated RMSE values are very close to 0, indicating that the calculation of the data is true and effective.

### 3.4. Perspectives and Prospects

In this study, a village with a complex distribution of buildings was selected to simulate the distribution of wind environment, and the steady-state solver most suitable for the village was selected. However, this does not represent all villages with a complex distribution of buildings. In the future research, we will continue to study as follows:

- More villages need to be selected and classified according to the characteristics of building layout, climate division, topographical conditions, number of buildings, and street size.
- Summarize the characteristics of villages presented by different classifications, use three solvers to calculate the same type of villages, and find out the relationship between similar villages and solvers.
- Summarize the calculation results and find the most suitable steady-state solver corresponding to different types of villages.

Subsequent research is also dedicated to finding the optimal steady-state solver, which will be more detailed, organized, and logical. It will provide a reference for the accuracy and reliability of the simulation of the village wind environment with a complex building layout. In the future, we still need to pay a lot of work to accomplish the above goals.

## 4. Conclusions

In this study, our main focus was the actual measurement and simulation of natural ventilation in a traditional Chinese village. The three steady-state simulations of CFD were compared in detail to find the steady-state solver that best simulates the wind environment of traditional Chinese villages to improve the accuracy of the simulated wind environment. Firstly, a detailed investigation was conducted on the research objects and the research area was determined. The study area was divided into four parts according to building density, and field measurements of wind-environment-related parameters were obtained in the four study areas. Secondly, Secondly, after comparing the measured value in the horizontal direction with the simulated value, it is concluded that SKE and RKE are more suitable for the simulation of a village wind environment with a complex building layout. The values generated by the simulation using the RNG solver are higher than the measured value. Through the local, whole village, and vertical direction, the reasons for the large deviation caused by the use of RNG were analyzed in detail. Finally, the horizontal and vertical directions are combined to analyze the reliability of the three solvers. Our conclusions are as follows:

- In the simulation of the village wind environment with a complex building layout, among the three steady-state solvers of FLUENT, the wind speed and turbulence intensity values obtained by the SKE solver have the highest reliability, and the degrees of fit are 0.8625 and 0.9088 respectively. The reliability of the RNG simulation is the lowest: the fit of the wind speed distribution is 0.7881, and the fit of the turbulence intensity is only 0.2473. Therefore, for villages with complex building layouts, the SKE solver should be the first choice when simulating wind speed distribution and turbulence intensity distribution.
- When using the RNG solver, the overall obtained turbulence intensity value is higher than the measured value. The simulated value at a height of 1.7 m differs from SKE and RKE by 42.61%. The main reason for this is that RNG over-represents the vortex and underestimates the airflow rate in the building interval.
- In the vertical direction, RNG cannot capture the complex wind flow structures that appear in the wake of high-rise buildings and narrow-span streets in complex building areas well, which leads to an overestimation of turbulence intensity values in these locations.

## Figures and Tables

**Table 1:** The detailed information of the instruments.

Instrument	Model	Precision	Measuring Range	Use
Hot wire anemometer	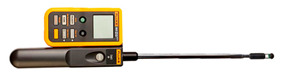	0.01 m/s	0.1–30 m/s	Measure wind speed
Infrared rangefinder	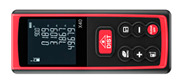	±1.0 mm	0.05–150 m	Measure distance

**Table 2:** The deviation between the measured and simulated average wind speed for different building densities.

	SKE	RNG	RKE
Density1 (37.14%)	9.74%	14.24%	13.39%
Density2 (35.71%)	20.53%	45.21%	26.26%
Density3 (35.58%)	3.28%	13.28%	21.43%
Density4 (24.76%)	20.35%	26.29%	12.74%
Mean Deviation	13.47%	24.76%	18.46%

**Table 3:** The deviation between the measured and simulated turbulence intensity for different building densities.

	SKE	RNG	RKE
Density1 (37.14%)	11.66%	8.52%	1.72%
Density2 (35.71%)	2.28%	17.04%	0.77%
Density3 (35.58%)	14.11%	17.28%	19.05%
Density4 (24.76%)	2.38%	6.48%	2.68%
Mean Deviation	7.61%	12.33%	6.06%

## Data Availability

Not applicable.
